# Ferromagnetic Nickel as a Sustainable Reducing Agent for Tin–Lead Mixed Perovskite in Single‐Junction and Tandem Solar Cells

**DOI:** 10.1002/advs.202411403

**Published:** 2024-12-12

**Authors:** Doyun Im, Passarut Boonmongkolras, Yeonghun Yun, Sung Woong Yang, Sunwoo Kim, Jungchul Yun, Rajendra Kumar Gunasekaran, You‐Hyun Seo, Nam Joong Jeon, Gill Sang Han, Sangwook Lee

**Affiliations:** ^1^ School of Materials Science and Engineering Kyungpook National University Daegu 41566 Republic of Korea; ^2^ Advanced Energy Materials Research Center Korea Research Institute of Chemical Technology (KRICT) Daejeon 34114 Republic of Korea; ^3^ Department Perovskite Tandem Solar Cells Helmholtz‐Zentrum Berlin für Materialien und Energie GmbH 12489 Berlin Germany

**Keywords:** all‐perovskite tandem solar cells, recyclable reducing agents, Sn–Pb mixed perovskites, standard reduction potentials

## Abstract

Narrow‐bandgap (NBG) Sn–Pb mixed perovskite solar cells (PSCs) represent a promising solution for surpassing the radiative efficiency of single‐junction solar cells. The unique bandgap tunability of halide perovskites enables optimal tandem configurations of wide‐bandgap (WBG) and NBG subcells. However, these devices are limited by the susceptibility of Sn^2+^ in the NBG bottom cell to being oxidized to Sn^4+^, creating detrimental Sn vacancies. Herein, a novel approach that replaces Sn particles with Ni particles is introduced as the reducing agent for Sn–Pb mixed perovskite precursor solutions. The ferromagnetic properties of Ni enable simple magnetic filtration, eliminating the filtration issues associated with Sn particles. Ni particles can be reused up to five times without significantly affecting the PSC's performance. Additionally, Ni effectively mitigates the oxidation of Sn^2+^ due to its low reduction potential (−0.23 V), thereby enhancing device performance. Single‐junction Sn–Pb mixed PSCs prepared using Ni achieve a power‐conversion efficiency (PCE) of 22.29%, retaining over 90% of their initial efficiency after 1250 h. Furthermore, Ni‐based all‐perovskite tandem solar cells combining 1.77 eV WBG top cells with 1.25 eV NBG bottom cells achieve a remarkable PCE of 28.13%. Thus, the proposed strategy can facilitate the commercialization of all‐perovskite tandem devices.

## Graphical abstract

Nickel is introduced as a more sustainable reducing agent for fabricating Sn–Pb mixed perovskite films. Due to its ferromagnetic properties, Ni allows for easy recycling. Ni effectively mitigates the oxidation of Sn^2+^ in the perovskite, resulting in enhanced performance. With the Ni reductant, single‐junction perovskite and all‐perovskite tandem solar cells achieved power conversion efficiencies of 22.29% and 28.13%, respectively, and demonstrated improved stability.

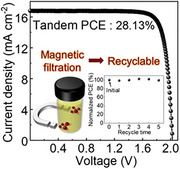

## 1 Introduction

Narrow‐bandgap Sn–Pb mixed perovskite solar cells (PSCs) have emerged as a crucial component in the development of high‐efficiency all‐perovskite tandem solar cells. These tandem devices offer a promising strategy to surpass the radiative efficiency of single‐junction solar cells and increase both their power‐conversion efficiency (PCE) and their weight‐specific power.^[^ ^1^, ^2^, ^3^, ^4^ ^]^ The unique bandgap tunability of halide perovskites allows for creating an optimal combination of wide‐bandgap (WBG, ≈1.77 eV) and narrow‐bandgap (NBG, ≈1.25 eV) subcells in all‐perovskite tandem configurations. Specifically, Sn–Pb mixed perovskites have demonstrated considerable potential as the NBG bottom cell material because it can lower the bandgap to ≈1.25 eV, which is suitable for high‐efficiency single‐junction devices and perovskite/perovskite tandem solar cells.^[^ ^5^, ^6^, ^7^ ^]^ Recent advancements in the development of Sn–Pb mixed PSCs have led to remarkable enhancements in all‐perovskite tandem device performance.^[^ ^8^, ^9^, ^10^, ^11^, ^12^, ^13^, ^14^ ^]^ For instance, a PCE of 28.5% has been achieved using a two‐terminal all‐perovskite tandem solar cell combining a 1.25 eV Sn–Pb NBG bottom subcell with a 1.78 eV WBG top subcell.^[^ ^15^ ^]^ This achievement underscores the critical role of Sn–Pb mixed perovskites in pushing the boundaries of solar cell efficiency.

However, the performance and stability of all‐perovskite tandem solar cells are primarily limited by the NBG Sn–Pb bottom cell. This limitation stems from the inherent instability of Sn^2+^, which easily oxidizes to Sn^4+^, creating Sn vacancies that increase nonradiative recombination and shorten the carrier lifetime.^[^ ^16^, ^17^ ^]^ These issues have instigated intensive research efforts to improve the quality and stability of Sn–Pb mixed perovskites.^[^ ^18^ ^]^ To mitigate the oxidation of Sn^2+^, various strategies have been explored, including i) compositional engineering of A‐site cations^[^ ^19^ ^]^ and B‐site cations^[^ ^20^, ^21^ ^]^ to identify perovskite compositions inherently less prone to oxidation, ii) surface passivation by introducing large A‐site cation‐based halide layers^[^ ^22^, ^23^, ^24^ ^]^ or regulating surface Sn–Pb–I ratios,^[^ ^25^ ^]^ iii) interfacial engineering by optimizing charge‐transport layers to minimize reactions with the perovskite layer,^[^ ^26^, ^27^ ^]^ iv) additive engineering using strong Lewis‐bases such as SnF_2_ and SnF_2_ coadditives (e.g., GuaSCN, FSA),^[^ ^28^, ^29^, ^30^ ^]^ and v) direct reduction of source materials by adding metallic particles to the perovskite precursor solution.^[^ ^31^ ^]^ The most widely adopted among these strategies has been the addition of SnF_2_ and/or metallic Sn to the perovskite precursor solution. However, SnF_2_ addition cannot entirely eliminate Sn^4+^ from the precursor solution, especially if DMSO and trace amounts of oxygen are present during the fabrication process.^[^ ^32^ ^]^ Meanwhile, R. Lin et al. reported that adding metallic Sn particles reduces Sn^4+^ to Sn^2+^ through the comproportionation reaction (Sn^0^ + Sn^4+^ → 2Sn^2+^),^[^ ^31^ ^]^ which significantly increases carrier lifetime and enhances the stability of both the perovskite precursor and the device.

Nevertheless, adding metallic Sn particles as a reducing agent necessitates a membrane filtration process, which is unsuitable for large‐scale usage; otherwise, unfiltered Sn particles can remain on the perovskite film surface, compromising the PCE by forming undesirable shunt pathways.^[^ ^33^ ^]^ To address these issues, we propose the use of metallic Ni particles as an alternative reducing agent. Ni, being a ferromagnetic metal, is amenable to a simpler magnetic filtration process. Moreover, as Ni has a lower reduction potential (−0.23 V) than Sn (−0.14 V),^[^ ^34^ ^]^ it ensures effective reduction of Sn^4+^ (Ni^0^ + Sn^4+^ → Ni^2+^ + Sn^2+^). Additionally, the small ionic radius (89 pm) of Ni^2+^ hinders its incorporation into the perovskite lattice, thereby minimally affecting the crystal structure of the perovskite thin films.

In this study, we experimentally demonstrated that metallic Ni can be efficiently filtered out from the perovskite precursor solution using a magnet, eliminating the problems caused by residual metallic particles. The Ni particles were reused up to five times without significantly compromising the photovoltaic (PV) performance of NBG PSCs. Additionally, metallic Ni effectively prevented undesirable disproportionation reactions, thereby enhancing device performance. Single‐junction Sn–Pb mixed PSCs prepared using metallic Ni achieved a PCE of 22.29% (aperture size of 0.094 cm^2^). Notably, devices with metallic Ni retained over 90% of their initial efficiency after 1250 h. Consequently, all‐perovskite tandem solar cells combining 1.77 eV WBG top cells with 1.25 eV NBG bottom cells prepared using metallic Ni as a reducing agent achieved a high PCE of 28.13%.

## 2 Results and Discussion

**Figure 1:**
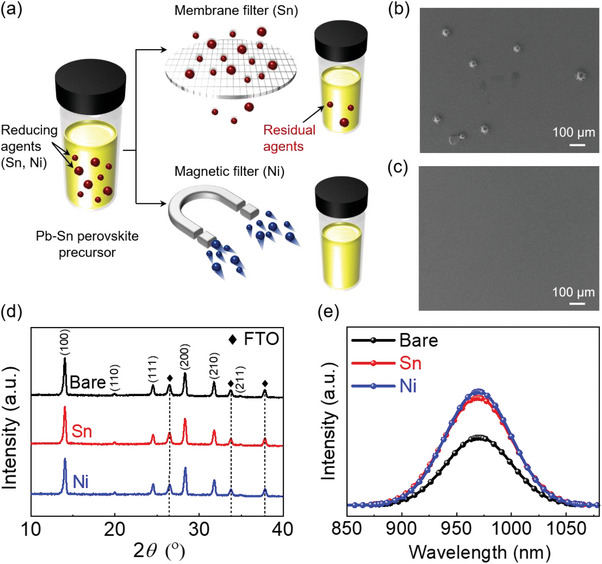
Comparison of filtration processes and fundamental properties of perovskite thin films without (Bare) and with reducing agents (Sn and Ni). a) Schematics illustrating membrane filtration and magnetic filtration processes. Top‐view scanning electron microscopy images of perovskite films b) using membrane filtration to remove Sn particles, showing residual unfiltered Sn particles, and c) using magnetic filtration to remove Ni particles, showing a clean surface without any unfiltered Ni particles. d) X‐Ray diffraction patterns of perovskite films without and with reducing agents. e) Photoluminescence spectra of perovskite films without and with reducing agents.

The perovskite precursor solutions were prepared using metallic Sn particles as a reducing agent and subsequently filtered through a 0.22 µm polytetrafluoroethylene (PTFE) membrane to remove the reducing agent particles, as shown in **Figure** **1**a. Despite this filtration process, Sn particles can remain in the filtered precursor solution because of their broad size distribution. (Figure S1, Supporting Information) The wide size distribution of Sn particles suggests that there are small particles, likely below the filtration limit, which could pass through the 0.22 µm membrane filter. Additionally, high filtration pressure during the process could further facilitate the passage of small Sn particles through the membrane. Residual Sn particles were observed on the perovskite film, as shown in Figure 1b. These particles can act as recombination sites and shunt pathways, resulting in poor device performance. Therefore, metallic Ni was chosen to replace metallic Sn as the reducing agent due to its low reduction potential and magnetic properties. Figure 1a illustrates the magnetic filtration process with Ni particles in the precursor solution, depicting the complete capture of Ni particles. Since magnetic process can filter particles irrespective of their size, no Ni particles were observed in the film despite the typically smaller size of Ni particles compared to Sn. (Figure 1c).

To confirm the effects of the reducing agents and filtration processes on the Sn–Pb mixed perovskite precursor solution, we characterized Sn–Pb mixed perovskite thin films without (Bare) and with the reducing agents (Sn and Ni). Figure 1d shows the X‐Ray diffraction (XRD) patterns of the Bare, Sn, and Ni samples, which exhibit no significant differences in peak shift and intensity. This indicates that the addition of the reducing agent did not alter the crystallographic orientation or phase of the Sn–Pb mixed perovskite thin films. The average grain sizes (≈650 nm) and thicknesses (≈900 nm) of the three perovskite thin film samples were fairly similar, as shown in Figures S2, S3 (Supporting Information); this is consistent with the XRD patterns in Figure 1d. Moreover, the UV–vis absorbance spectra of the perovskite thin films showed no significant difference under any condition, and the optical bandgap energies were calculated to be 1.25 eV using a Tauc plot, as shown in Figure S4 (Supporting Information). However, the steady‐state photoluminescence (PL) spectra in Figure 1e show that the PL peak intensity increases with the addition of reducing agents to the precursor. This result indicates that these agents effectively limit nonradiative recombination in the resulting perovskite film, probably due to the reduction of defect formation related to the oxidation of Sn^2+^ ions. In addition, the peak intensity of the Ni sample was slightly higher than that of the Sn sample. The PL peak position is slightly (≈0.02 eV) blue shifted, compared to the bandgap (1.25 eV) determined from the absorbance spectrum, which is attributed to the effect of hot carrier relaxation that is known to be slow in tin‐based perovskites.^[^ ^35^ ^]^

**Figure 2:**
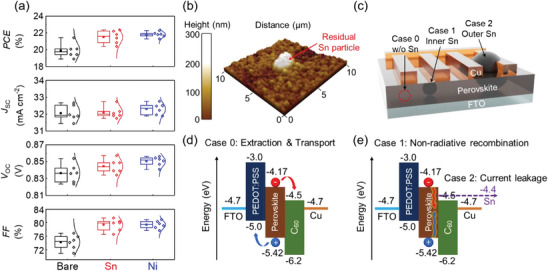
Comparison of photovoltaic performance of PSCs without (Bare) and with the agents (Sn and Ni). a) Statistical distributions of PV parameters of single‐junction solar cells. b) 3D atomic force microscopy image depicting remaining Sn particle on perovskite film surface. c) Schematic of three cases without and with (inner, outer) metal particle in the PSC. d) Schematic illustrating charge carrier extraction and transport in PSC without residual metal particle. e) Schematic showing recombination and current leakage in PSC caused by the residual Sn particle.

To examine the effects of Ni as a reducing agent on device performance, we fabricated Sn–Pb mixed PSCs with a fluorine‐doped tin oxide (FTO)/poly(3,4‐ethylenedioxythiophene):polystyrenesulfonate (PEDOT:PSS)/ FA_0.6_MA_0.4_Sn_0.6_Pb_0.4_I_3_ (NBG perovskite)/ethane‐1,2‐diammonium iodide (EDAI_2_)/C_60_/bathocuproine (BCP)/Cu structure. **Figure** **2**a and **Table** **1** present the statistical distributions of the PV parameters for each sample. The average short‐circuit current density (*J*_SC_) and its distribution were similar across all devices, consistent with the grain size, optical bandgap, crystallinity, and thickness of the perovskite film in each sample. However, the open‐circuit voltage (*V*_OC_) and fill factor (FF) increased upon the addition of the reducing agent. The average performance of the Sn‐based device was similar to that of the Ni‐based device, while the Sn‐based device exhibited a larger efficiency distribution due to a wider distribution of *V*_OC_ and FF. As shown in Figure S5 (Supporting Information), additional filtration can improve device performance and reduce the distribution of photovoltaic parameters. This result supports our conclusion that the membrane filtration does not perfectly filter the Sn particles, which affects the device performance.

**Table 1:** Average performance of single‐junction solar cells.

	*J*_SC_ [mA cm^−2^]	*V*_OC_ [V]	FF [%]	PCE [%]
Bare	32.06 ± 0.42	0.836 ± 0.010	74.19 ± 1.96	19.90 ± 0.80
Sn	32.09 ± 0.32	0.845 ± 0.007	79.32 ± 1.63	21.50 ± 0.71
Ni	32.30 ± 0.31	0.850 ± 0.005	79.38 ± 1.09	21.80 ± 0.30

As mentioned previously, residual Sn particles can negatively impact device performance. These particles are clearly observable in the atomic force microscopy (AFM) topographical image in Figure 2b. The Sn particles resulted in a high surface roughness (RMS = 127.73 nm) compared with that of the smooth perovskite surface (RMS = 36.76 nm), as shown in Figure S6 (Supporting Information). Figure 2c schematically illustrates the effects of the residual Sn particles on the PSC. Depending on their size, the residual Sn particles may be embedded within the perovskite film or remain on its surface. Figure 2d,e shows the energy levels configuration of the p–i–n–structured Sn–Pb mixed PSCs.^[^ ^36^ ^]^ The conduction band minimum of the Sn–Pb mixed perovskite film was at −4.17 eV, while the valence band maximum was at −5.42 eV, which was obtained from ultraviolet photoelectron spectroscopy measurement as shown in Figure S7 (Supporting Information). However, the work function of metallic Sn is ≈−4.4 eV,^[^ ^37^, ^38^ ^]^ hence, the residual Sn particles are likely to degrade the device performance by acting as nonradiative recombination sites (Case 1, indicated in Figure 2c) and shunt pathways (Case 2), leading to a wide distribution of *V*_OC_ and FF. In contrast, the magnetic filtration process allowed the Ni‐based sample to operate without any performance loss, resulting in a narrow distribution of PCE, as shown in Figure 2e.

**Figure 3:**
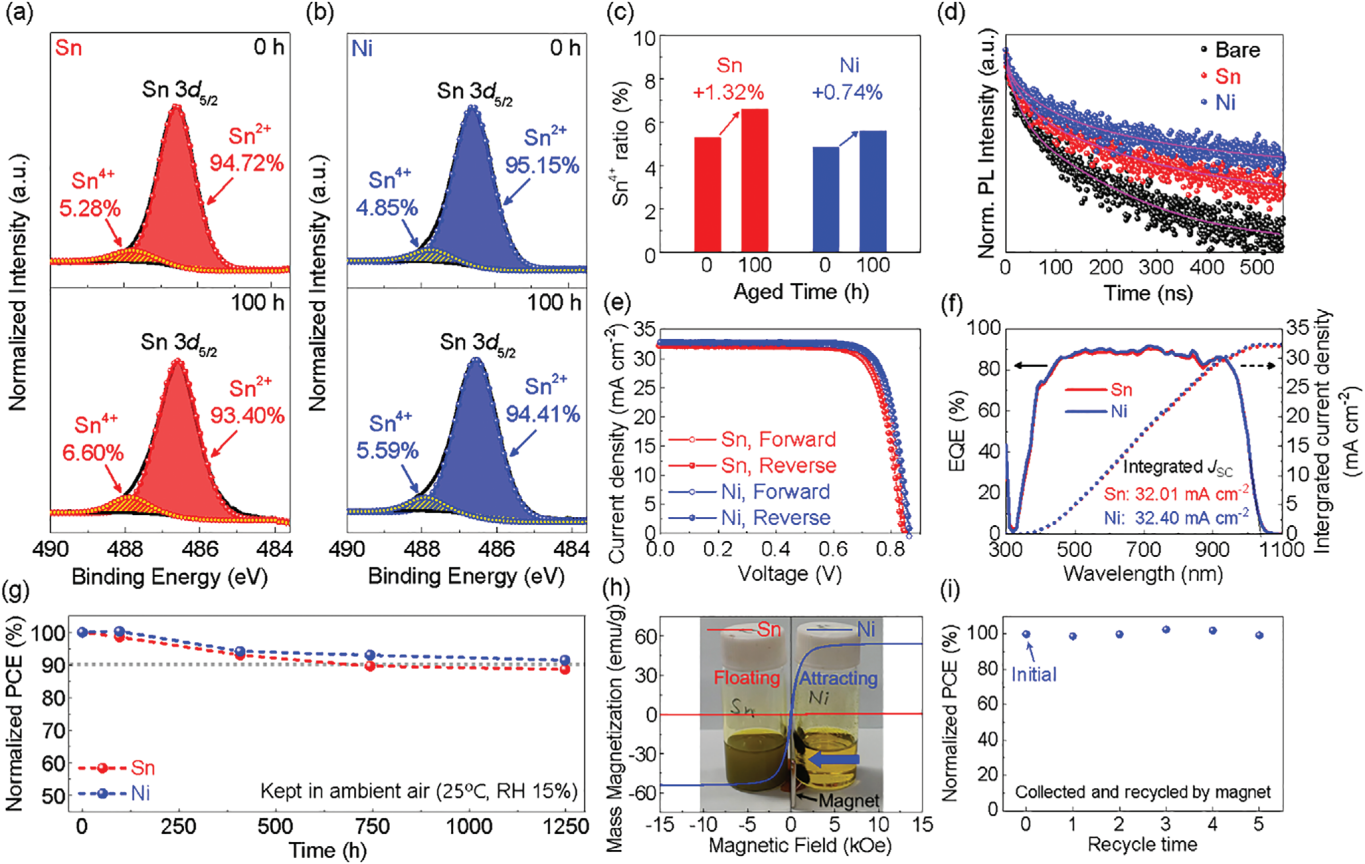
Analyses of perovskite thin films and performance of single‐junction solar cells prepared using metallic Sn and Ni as a reducing agent. X‐Ray photoelectron spectroscopy of perovskite films with a) metallic Sn and b) Ni as a reducing agent. c) Sn^4+^ ratio of Sn 3*d*_5/2_ peak for 0 and 100 h aged perovskite films with Sn and Ni. d) Time‐resolved PL of Bare, Sn‐based, and Ni‐based perovskite films with the sample configuration of FTO/PEDOT:PSS/perovskite. e) *J–V* curves of single‐junction Sn‐ and Ni‐based PSCs. f) External quantum efficiency and integrated current density of Sn‐ and Ni‐based PSCs. g) Long‐term stability of Sn‐ and Ni‐based PSCs. h) *M–H* curves of metallic Sn and Ni particles and (inset) photograph of perovskite precursor solutions with metallic Sn and Ni. i) Normalized PCE of PSCs after recycling the metallic Ni five times.

To compare the reducing effects between Sn and Ni particles on the Sn–Pb mixed perovskite precursor, we conducted X‐Ray photoelectron spectroscopy (XPS) analysis on a perovskite thin film with a composition of FA_0.6_MA_0.4_Sn_0.6_Pb_0.4_I_3_. **Figure** **3**a,b respectively show the binding energies of the Sn 3*d*_5/2_ peak for the Sn‐based and Ni‐based Sn–Pb mixed perovskite thin films, comparing a fresh sample and a 100‐h‐aged sample in each case. It should be noted that we made efforts to minimize the residual metallic Sn particles by multiple filtrations to ensure accurate analysis of Sn oxidation states, which explains the absence of Sn^0^ peak in the XPS spectra. The Sn^4+^ ratio of the Ni sample was 0.43 percentage point less than that of the Sn sample for the fresh perovskite films. Since perovskite precursor solutions are filtered immediately before the spin‐coating, it is difficult to expect any dramatic reduction from residual Sn particles at this stage. Interestingly, this gap widened to 1.01 percentage point for the films aged for 100 h. After 100 h, the Sn^4+^ ratio of the Sn and Ni samples increased as much as 1.32 and 0.74 percentage point, respectively, as shown in Figure 3c. This indicates that metallic Ni can efficiently retard the oxidation of Sn^2+^ in the perovskite precursor solution.

Figure S8 and Table S1 (Supporting Information) show a Frost diagram and standard reduction potentials explaining the propensity of Sn for oxidation. The Frost diagram for Pb has a deep asymmetric thermodynamic sink, demonstrating the stability of the Pb^2+^ oxidation state. By comparison, Sn^2+^ shows a very shallow and symmetric thermodynamic sink, which creates several undesired pathways toward higher and lower oxidation states (i.e., Sn^4+^ or Sn^0^). In contrast, metallic Ni (Ni^0^) shows a deep asymmetric sink similar to that of Pb, in addition to having a lower standard reduction potential than Sn^0^. This indicates that Ni can provide a stable Sn–Pb mixed perovskite precursor due to its energetically lower reduction potential than Sn. Additionally, the Sn–Pb mixed perovskite film did not display any Ni peak in its XPS pattern, as observed in Figure S9 (Supporting Information).

The effects of oxidation on the charge recombination time and trap density of Sn–Pb mixed perovskite films were characterized through time‐resolved PL (TRPL) and space charge–limited current (SCLC) analyses, as shown in Figure 3d and Figure S10 (Supporting Information), respectively. The TRPL analysis revealed that the average recombination time (Bare = 37.04 ns, Sn = 71.97 ns, Ni = 104.70 ns), which is represented in Table S2 (Supporting Information), increased with the addition of the reducing agent. Specifically, the addition of Ni as a reducing agent ensured a longer carrier lifetime compared with the addition of Sn. The trap densities were determined to be 2.18 × 10^15^, 1.54 × 10^15^, and 1.41 × 10^15^ cm^−3^ for the Bare, Sn, and Ni samples, respectively, as shown in Figure S10 (Supporting Information). These results indicate that successful retardation of Sn^2+^ oxidation by the Ni reducing agent mitigated the nonradiative recombination and reduced the trap density in the Sn–Pb mixed perovskite film.

Figure 3e shows the *J–V* curve of the representative devices, which were fabricated with Sn and Ni as the reducing agent. Ni‐based (Sn‐based) device achieved a high PCE of 22.29 (21.81)%, with a *J*_SC_ of 32.48 (32.16) mA cm^−2^, a *V*_OC_ of 0.854 (0.849) V, and an FF of 80.32 (79.93)%. In addition, the integrated current densities of Sn‐ and Ni‐based devices, derived from the external quantum efficiency (EQE), were 32.01 and 32.40 mA cm^−2^, respectively (Figure 3f), which is consistent with the *J*_SC_ from the *J–V* measurement. Figure 3g shows the long‐term stability of the Sn‐ and Ni‐based device. After undergoing encapsulation, the Ni‐based device maintained over 90% of its initial PCE for 1250 h (T_90_ > 1250 h), while the Sn‐based device maintained 90% of its initial PCE for 750 h (T_90_ = 750 h), in a dry room (air ambient conditions, room temperature, 15% relative humidity). As mentioned earlier, Ni particles can be easily collected from the Sn–Pb mixed perovskite precursor using a magnet. Ni exhibits a high saturation magnetization (*M*_s_) of over 50 emu g^−1^, whereas Sn does not possess any magnetic properties, as shown in Figure 3h. Additionally, if Sn is discarded into water after being used as a reducing agent, it can negatively impact the environment through phenomena such as soil acidification.^[^ ^39^ ^]^ By contrast, Ni can be recycled using a magnetic filter. To confirm the recyclability of Ni particles, Sn–Pb mixed PSCs were repeatedly fabricated by reusing the filtered Ni particles five times. Figure 3i shows the PCEs of the fabricated devices, which do not exhibit any significant difference. These findings strongly suggest that Ni is a promising reducing agent material to replace Sn because it enables the preparation of a highly pure Sn–Pb perovskite precursor by removing residual Ni particles using a magnetic filter, retards the oxidation of Sn^2+^ due to its low reduction potential, and can be reused for the fabrication of multiple PSCs.

**Figure 4:**
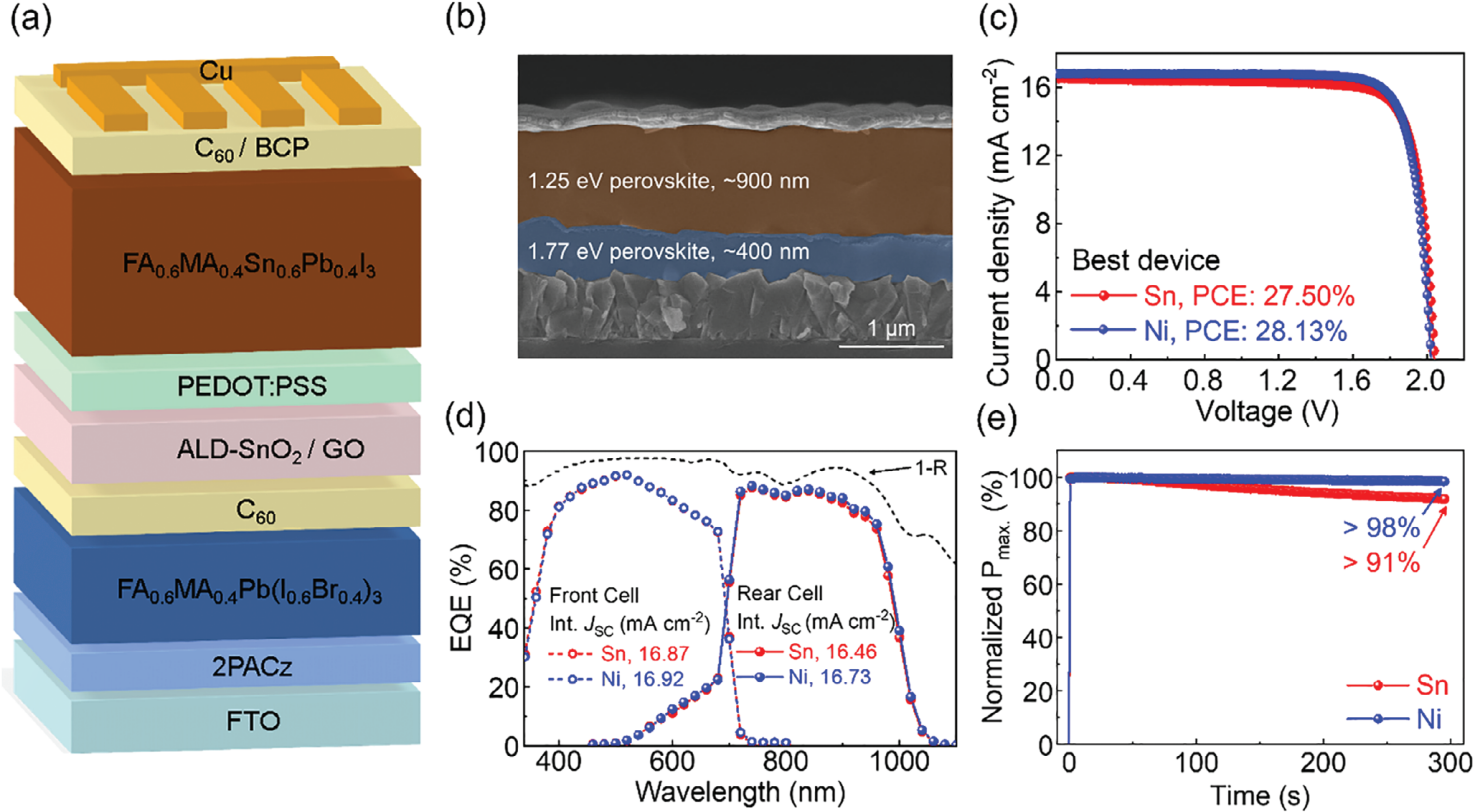
Monolithic all‐perovskite tandem solar cells fabricated using metallic Ni as a reducing agent of Sn‐Pb perovskite precursor. a) 3D structure of monolithic all‐perovskite tandem solar cell. b) Cross‐sectional scanning electron microscopy image of all‐perovskite tandem solar cell. c) *J–V* curves of all‐perovskite tandem solar cell fabricated using Sn and Ni as reductant. d) EQE of Sn‐ and Ni‐based tandem solar cell with 1‐R. e) Stabilized power output of Sn‐ and Ni‐based tandem device for 300 s.

We fabricated monolithic all‐perovskite tandem devices with a (FTO/[2‐(9H‐Carbazol‐9‐yl)ethyl]phosphonic acid (2PACz)/FA_0.6_MA_0.4_Pb(I_0.6_Br_0.4_)_3_ (WBG perovskite)/C_60_/ALD‐SnO_2_/graphene oxide (GO)/PEDOT:PSS/NBG perovskite/C_60_/BCP/Cu) structure, as shown in **Figure** **4**a. A 1.25 eV NBG PSC acting as a rear subcell (≈900 nm thick) was stacked on a 1.77 eV WBG PSC acting as a front subcell (≈400 nm thick) (Figure 4b). As a reference, we used a single‐junction WBG PSC, which exhibited a PCE of 18.76% under reverse scan, as shown in Figure S11 and Table S3 (Supporting Information). Figure 4c shows the *J–V* curves of the best‐performing tandem device using Sn and Ni as reductant under reverse scan. The Ni‐based tandem device achieved a maximum PCE of 28.13% with a *J*_SC_ of 16.81 mA cm^−2^, a *V*_OC_ of 2.038 V, and an FF of 82.10%, as shown in **Table** **2**. The integrated current densities of the front and rear cells of Ni‐based (Sn‐based) tandems were 16.92 (16.87) mA cm^−2^ and 16.73 (16.46) mA cm^−2^, respectively, which is in close agreement with the *J*_SC_ from the *J–V* curves of tandem devices, as shown in Figure 4d. The Ni‐based tandem device exhibited an average PCE of 27.11 ± 0.53%, as shown in Figure S12 (Supporting Information), and Table 2. In addition, operational stability was evaluated with a stabilized power output under 1‐sun illumination in ambient air; based on the results, the Ni‐based (Sn‐based) tandem device maintained ≈98 (91)% of its initial PCE for 300 s (Figure 4e).

**Table 2:** Average and best device performance of all‐perovskite tandem solar cells using Sn and Ni as reductant.

	*J*_SC_ [mA cm^−2^]	*V*_OC_ [V]	FF [%]	PCE [%]
Sn (Average)	16.32 ± 0.32	2.012 ± 0.028	81.81 ± 1.74	26.86 ± 0.46
Ni (Average)	16.39 ± 0.37	2.022 ± 0.026	81.81 ± 1.36	27.11 ± 0.53
Sn (Best)	16.54	2.042	81.42	27.50
Ni (Best)	16.81	2.038	82.10	28.13

## 3 Conclusion

Herein, we introduced a novel approach for the fabrication of Sn–Pb mixed PSCs that involves using metallic Ni as a reducing agent instead of Sn. By leveraging the ferromagnetic nature of Ni, residual metallic particles can be removed from the perovskite precursor through a simple magnetic filtration process, unlike the conventional membrane filtration process required for Sn. Moreover, the filtered Ni particles can be reused for further fabrication without affecting the performance of the PSC significantly. Additionally, owing to its low standard reduction potential, Ni can effectively reduce the Sn^4+^ in the perovskite film, thereby mitigating nonradiative recombination and lowering the defect density. Single‐junction PSCs prepared using Ni achieved enhanced performance and a narrow PCE distribution; the highest PCE attained was 22.29%, and 90% of the initial PCE was maintained after 1250 h. Furthermore, all‐perovskite tandem solar cells fabricated using Ni exhibited a maximum PCE of 28.13%. Therefore, by ensuring adequate material recyclability, the proposed strategy provides a sustainable route for the commercialization of all‐perovskite tandem solar cells.

## Conflict of Interest

The authors declare no conflict of interest.

## Supporting information



Supporting Information

## Data Availability

The data that support the findings of this study are available from the corresponding author upon reasonable request.
