# Supplementary material for: Ferromagnetic Nickel as a Sustainable Reducing Agent for Tin–Lead Mixed Perovskite in Single‐Junction and Tandem Solar Cells
Source: Adv Sci (Weinh). 2024 Dec 12;12(5):2411403. doi: 10.1002/advs.202411403 (PMC11791981; doi:10.1002/advs.202411403)
Supplement: Supplementary file 1 — Supporting Information [file ADVS-12-2411403-s001.docx]

Supporting Information

**Ferromagnetic Nickel as a Sustainable Reducing Agent for Tin–Lead Mixed Perovskite in Single-Junction and Tandem Solar Cells**

Doyun Im^1^, Passarut Boonmongkolras^2^, Yeonghun Yun^3^, Sung Woong Yang^1^, Sunwoo Kim^1^, Jungchul Yun^1^, Rajendra Kumar Gunasekaran^1^, You-Hyun Seo^2^, Nam Joong Jeon^2^, Gill Sang Han^2*^, Sangwook Lee^1*^

D. Im, S. W. Yang, S. Kim, J. Yun, R. K. Gunasekaran, S. Lee

^1^School of Materials Science and Engineering, Kyungpook National University, Daegu 41566, Republic of Korea
E-mail: wook2@knu.ac.kr

P. Boonmongkolras, Y.-H. Seo, N. J. Jeon, G. S. Han
^2^Advanced Energy Materials Research Center, Korea Research Institute of Chemical Technology (KRICT), Daejeon 34114, Republic of Korea

E-mail: gshan@krict.re.kr

Y. Yun

^3^Department Perovskite Tandem Solar Cells, Helmholtz-Zentrum Berlin für Materialien und Energie GmbH, 12489 Berlin, Germany

Experimental Section

*Materials:* All the chemicals and solvents were used without any additional processing. CsI (99.9%), SnF_2_ (99%), Sn metal (99.5%), Ni metal (99.99%), ethane-1,2-diammonium iodide (EDAI_2_, 98%), ethylenediamine (EDA, 99%), N,N-dimethylformamide (DMF, 99.8%, anhydrous), dimethyl sulfoxide (DMSO, 99.9%, anhydrous), ethyl acetate (EA, 99.8%, anhydrous), chlorobenzene (CB, 99.8%, anhydrous), and isopropyl alcohol (IPA, 99.5%, anhydrous) were purchased from Sigma-Aldrich. Formamidinium iodide (FAI, 99.99%), methylammonium iodide (MAI, 99.99%), and cobalt (III) bis(trifluoromethanesulfonyl)imide (Co-TFSI) were purchased from Greatcell Solar. PbI_2_ (99.999%, anhydrous beads) and SnI_2_ (99.999%, anhydrous beads) were purchased from Alfa Aesar. GuaSCN (>99.0%), PbBr_2_ (>98.0%), PbI_2_ (>98.0%), [2-(9H-Carbazol-9-yl)ethyl]phosphonic acid (2PACz, >98.0%), and BCP (>99.0%) were purchased from Tokyo Chemical Industry Co., LTD (TCI, Japan). PEDOT:PSS aqueous solution (CLEVIOS P VP AI 4083) was purchased from Heraeus, LLC. C_60_ (99.5%) was purchased from 1-Material. 2,2,7,7-Tetrakis(N,N-di-p-methoxyphenylamine)-9,9-spirobifluorene (Spiro-OMeTAD, 99.5%) was purchased from Luminescence Technology Corporation. Graphene oxide was purchased from graphene supermarket. Tetrakis(dimethylamino) tin (IV) (TDMASn, 99.9%) was purchased from EGChem Co., Ltd. Cu and Au pellets (3 mm diameter) were purchased from iTASCO.

*Precursor Solution Preparation:* The 1.25 eV NBG perovskite precursor solution was prepared in a 1.8 M concentration by mixing FAI/MAI and SnI_2_/PbI_2_ in molar ratios of 0.6:0.4 and 0.6:0.4, respectively, in a mixed solvent containing DMF and DMSO in a 4:1 volume ratio. CsI (5 mol% in excess), SnF_2_ (10 mol% relative to SnI_2_), and GuaSCN (5 mol% relative to MAI) were added to the precursor solution and stirred overnight at room temperature and filtered through a 0.22 μm PTFE membrane. Then, the filtered solution was separately moved into individual vials of 3 mol% as-prepared metallic Sn and Ni particles relative to the perovskite (ABX_3_). After 10 min of stirring at 1000 rpm using a vortex mixer, the reduced precursor solution was re-filtered by membrane and magnetic filtration processes.

The 1.77 eV WBG perovskite precursor solution was prepared following the typical double-cation process. In brief, 1.15 M stock solution was prepared by mixing PbI_2_, PbBr_2_, FAI, and MABr in a mixed solvent containing DMF and DMSO in a 4:1 volume ratio to form a FA_0.6_MA_0.4_Pb(I_0.6_Br_0.4_)_3_ perovskite composition. Subsequently, 10% excess PbI_2_ was added to the precursor solution. The solution was thoroughly mixed overnight and filtered through a 0.22 µm PTFE membrane before spin-coating.

*Magnetic filtration process and Ni metal recycling:* The magnetic filtration was conducted by applying magnetic field with a commercially available Nd magnet (Residual flux density = ~1.4 T). To prevent the Ni particles from participating in additional reactions before reuse, DMF was added into the vial containing the already used Ni particles with the residual perovskite precursor solution inside of the glove box. The DMF-containing vial was briefly stirred then DMF was removed, while the Ni particles were magnetically captured in the vial for recycle.

*Device Fabrication:* The single-junction 1.25 eV NBG PSCs were fabricated using pre-patterned FTO glass with dimensions of 2.5 × 2.5 cm^2^ as the substrate. The substrates were sequentially cleaned via sonication with a detergent (Hellmanex solution), acetone, deionized water, and IPA, each for 3 min, before being dried using a nitrogen stream. Thereafter, the surface of the FTO was treated with a UV-ozone cleaner for 20 min. PEDOT:PSS was then spin-coated onto the UV-ozone-treated FTO substrates at 5,000 rpm for 30 s, following which the substrates were annealed at 120 °C for 20 min in ambient air. The samples were transferred to an argon-filled glove box after being cooled. Then, 100 µL of the NBG perovskite precursor solution was dropped on the PEDOT:PSS-coated samples, which were spun at 1000 rpm for 10 s and then at 4000 rpm for 40 s, with an acceleration of 1000 rpm∙s^-1^. Subsequently, 200 µL of EA was dropped on the spinning substrate 20 s before the end of spin-coating. The substrates were then annealed at 100 °C for 10 min. Next, they were spin-coated at 4000 rpm for 30 s with an EDAI_2_ solution prepared by dissolving 1 mg EDAI_2_ powder in 1 mL IPA, before being annealed again at 100 °C for 5 min. After being cooled, the substrates were transferred to a thermal evaporation chamber for the deposition of 20 nm C_60_, 5 nm BCP, and 100 nm Cu. Finally, the devices were encapsulated with UV epoxy and cover glass for characterization.

To fabricate the single-junction 1.77 eV WBG PSCs, the FTO substrates were transferred to the glove box after UV–ozone treatment. A 2PACz solution (1 mM) prepared by dissolving 2PACz powder in IPA was spin-coated onto the FTO substrate at 3000 rpm for 30 s, whereafter the substrates were annealed at 100 °C for 10 min. Then, 100 µL of the WBG perovskite solution was spin-coated onto the FTO/2PACz substrate at 6000 rpm for 30 s. Next, 200 µL of CB was dropped onto the center of the film 25 s before the end of spin-coating. The perovskite samples were then annealed at 100 °C for 20 min, after which they were spin-coated with 100 µL of EDA solution (0.1 mM EDA in toluene) at 3000 rpm for 30 s, before being annealed again at 65 °C for 5 min. After being cooled, the substrates were transferred to the thermal evaporation chamber for the deposition of 20 nm C_60_, 5 nm BCP, and 100 nm Cu.

To fabricate the all-perovskite tandem solar cells, after the deposition of C_60_ on top of the WBG perovskite layer, the samples were extracted and transferred to an atomic layer deposition system (CN1, Atomic-Classic). Then, 25 nm SnO_2_ was deposited using a tetrakis(dimethylamino) tin (IV) precursor and deionized water at 90 °C. Thereafter, graphene oxide was spin-coated on the SnO_2_ layer. The subsequent processes were identical to the process followed for the single-junction NBG device.

*Device Characterization:* The device characterizations were performed under ambient air after encapsulating the devices. The *J*–*V* characteristics were measured using a source meter (Keithley 2450, Keithley), from forward bias to reverse bias, at a scan rate of 100 mV s^−1^. A solar simulator (Oriel Solar 3A Class, 94023A, Newport), which was calibrated to illuminate AM 1.5G (100 mW cm^−2^) using a standard Si reference cell (91150V, Newport), was utilized. The illuminated active area of the device was 0.094 cm^2^. The steady-state PCEs were measured using a potentiostat (PGSTAT204, Autolab). The hole-only device configuration for the SCLC analysis was FTO/PEDOT:PSS/perovskite/Spiro-OMeTAD/Au, which was measured with a source meter (Keithley 4200-SCS, Keithley), and calculated based on a previous report. ^[1]^ The EQE spectra of the single-junction NBG devices and the all-perovskite tandem devices were measured using a tunable light source for QE (TLS-300XU, Newport).

*Other characterizations:* XRD analysis was performed using an X'Pert instrument from PANalytical with Cu Kα beams. The surface morphology and cross-sections were observed using a field-emission scanning electron microscope (JSM-6701F, JEOL). The absorption and reflectance spectra of the perovskite thin films were obtained via UV–Vis–NIR spectroscopy (Cary 5000, Agilent Technologies). Integrating sphere was used to obtain the reflectance spectra. The steady-state PL was measured using a custom-built setup with a 532 nm diode laser (Thorlabs) and an Ocean Optics Spectrometer (FLAME-T model). The TRPL was measured by using a lifetime fluorometer (FluoTime 250, PicoQuant), which is equipped with a 470 nm pulsed laser using 700 nm longpass filter with a time-correlated single photon counting (TCSPC) technique. The residual Sn particles were observed via AFM (NX10, Park systems). The surface chemical states of the perovskite films were analyzed via XPS (Theta Probe AR-XPS System, Thermo Fischer Scientific). The work function and valence band maximum of the perovskite film were obtained using ultraviolet photoelectron spectroscopy (UPS, AXIS NoVA, Kratos) with a He-I line lame (21.22 eV). The magnetic properties of the Sn and Ni particles were analyzed using a vibrating sample magnetometer (VSM, 7407-S, LakeShore) at the Kyungpook National University Center for Research Facilities.


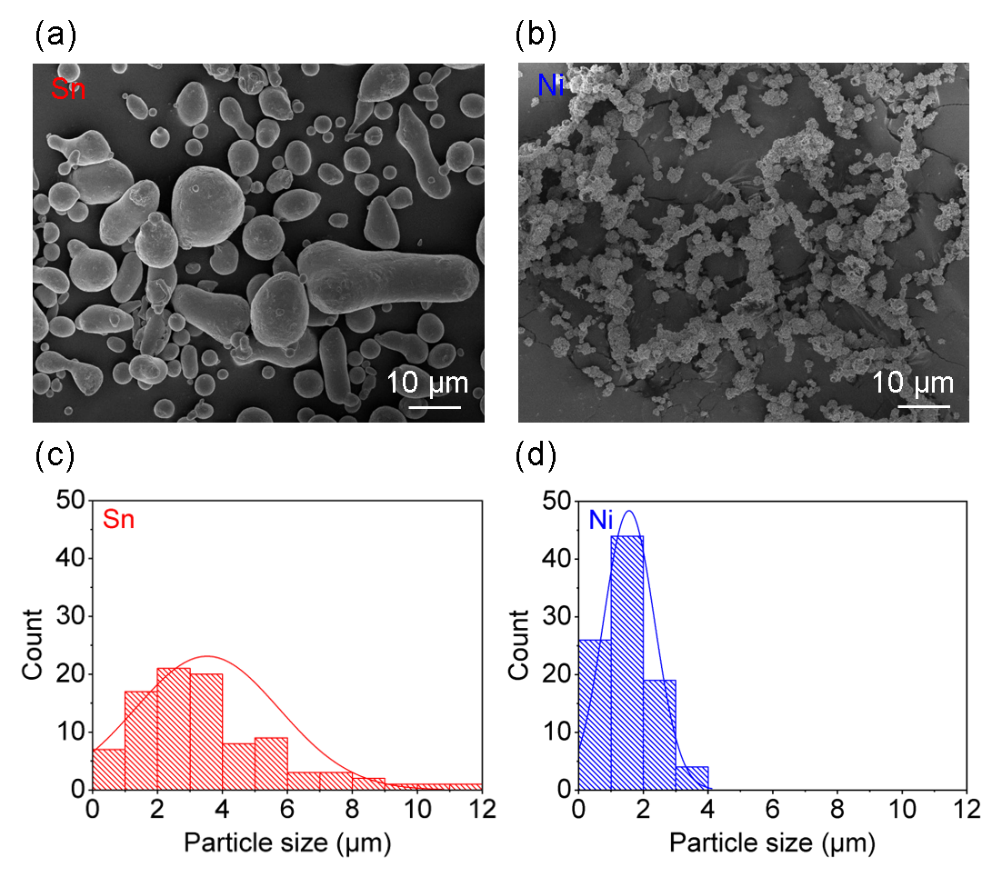


**Figure S1.** Scanning electron microscope images of (a) Sn and (b) Ni particles. Particle size distribution histograms of (c) Sn and (d) Ni.


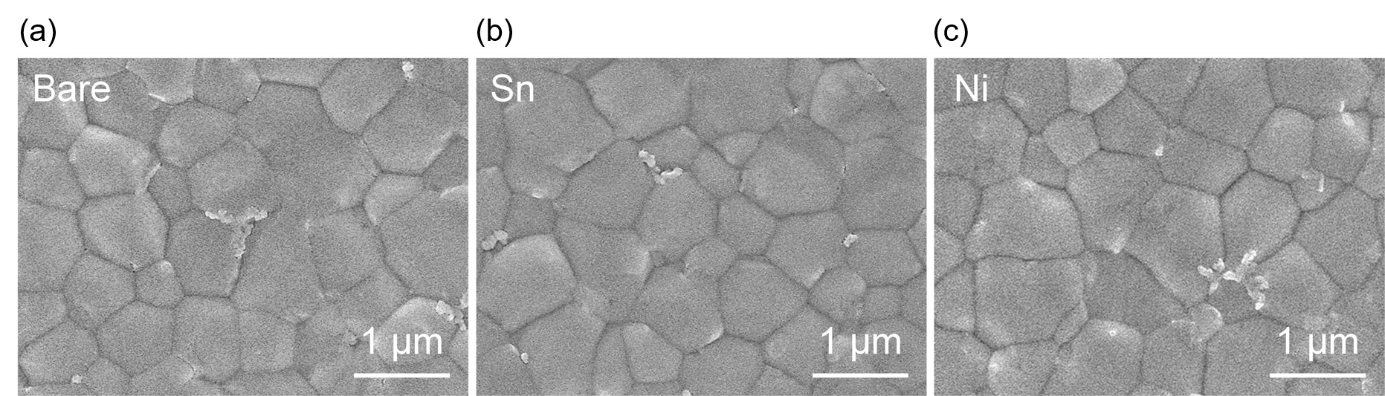


**Figure S2.** Top-view scanning electron microscope images of (a) Bare, (b) Sn-based, and (c) Ni-based perovskite films.


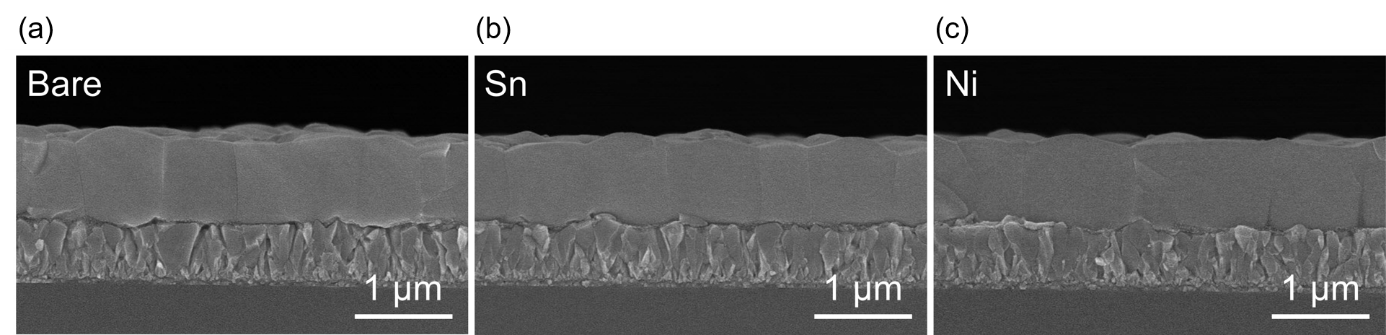


**Figure S3.** Cross-sectional scanning electron microscope images of (a) Bare, (b) Sn-based, and (c) Ni-based perovskite films.


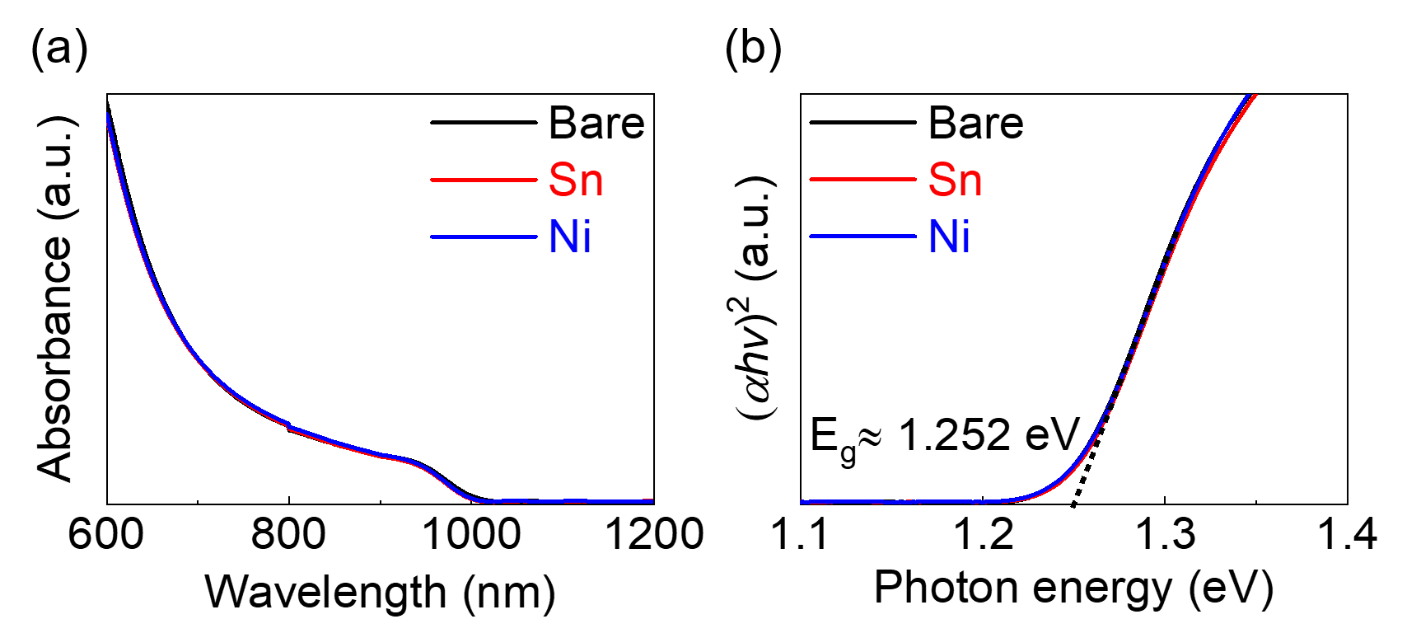


**Figure S4.** UV–vis absorbance spectra of (a) Bare, (b) Sn-based, and (c) Ni-based perovskite films.


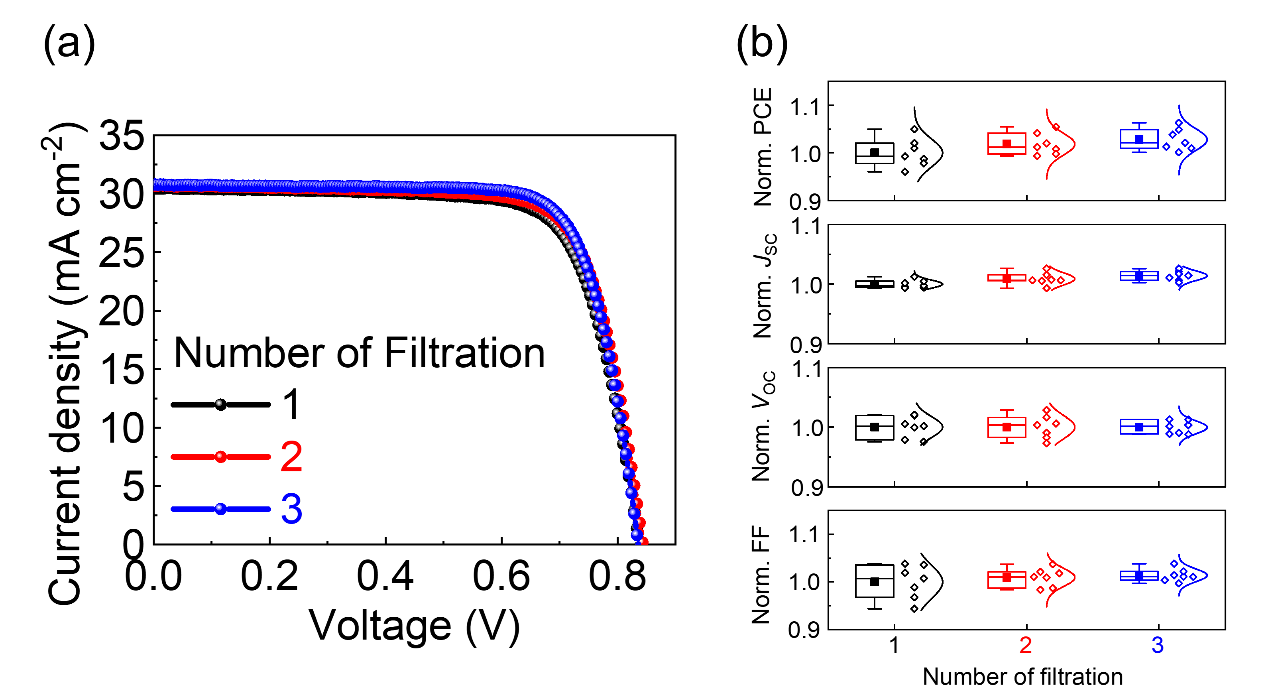


**Figure S5.** (a) *J–V* curves and (b) statistical distributions of PV parameters of Sn-based single-junction devices based on the number of filtration.


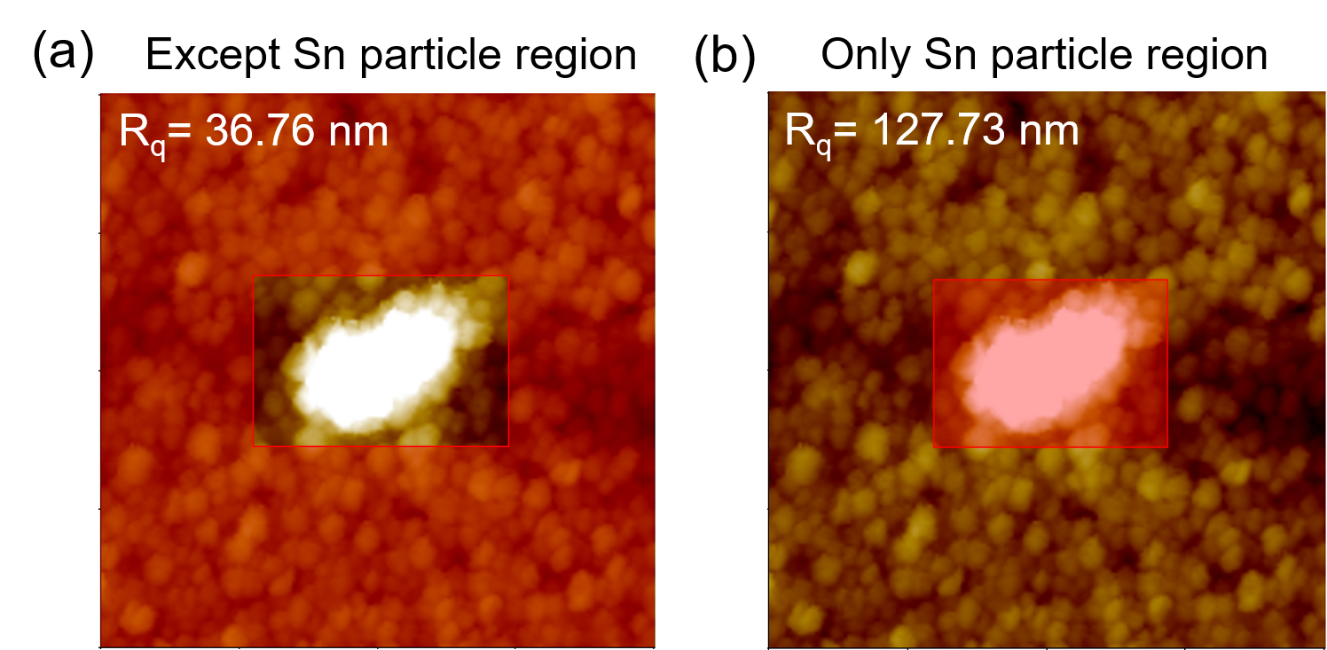


**Figure S6.** Atomic force microscopy image of perovskite film with residual Sn particle at the center. Calculated root-mean-square roughness of film surface (a) except the Sn particle region and (b) containing only the Sn particle region.


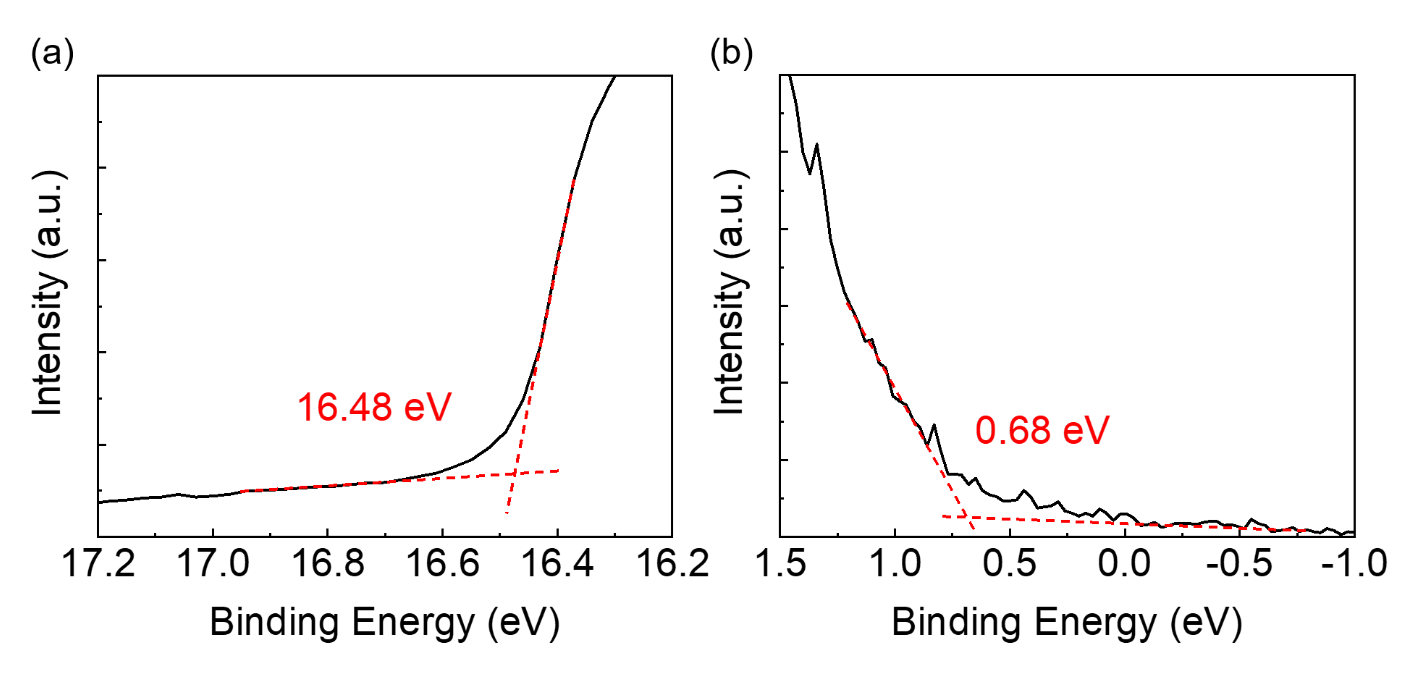


**Figure S7.** Ultraviolet photoelectron spectroscopy analysis of perovskite film: (a) secondary electron cut-off region and (b) valence band region.


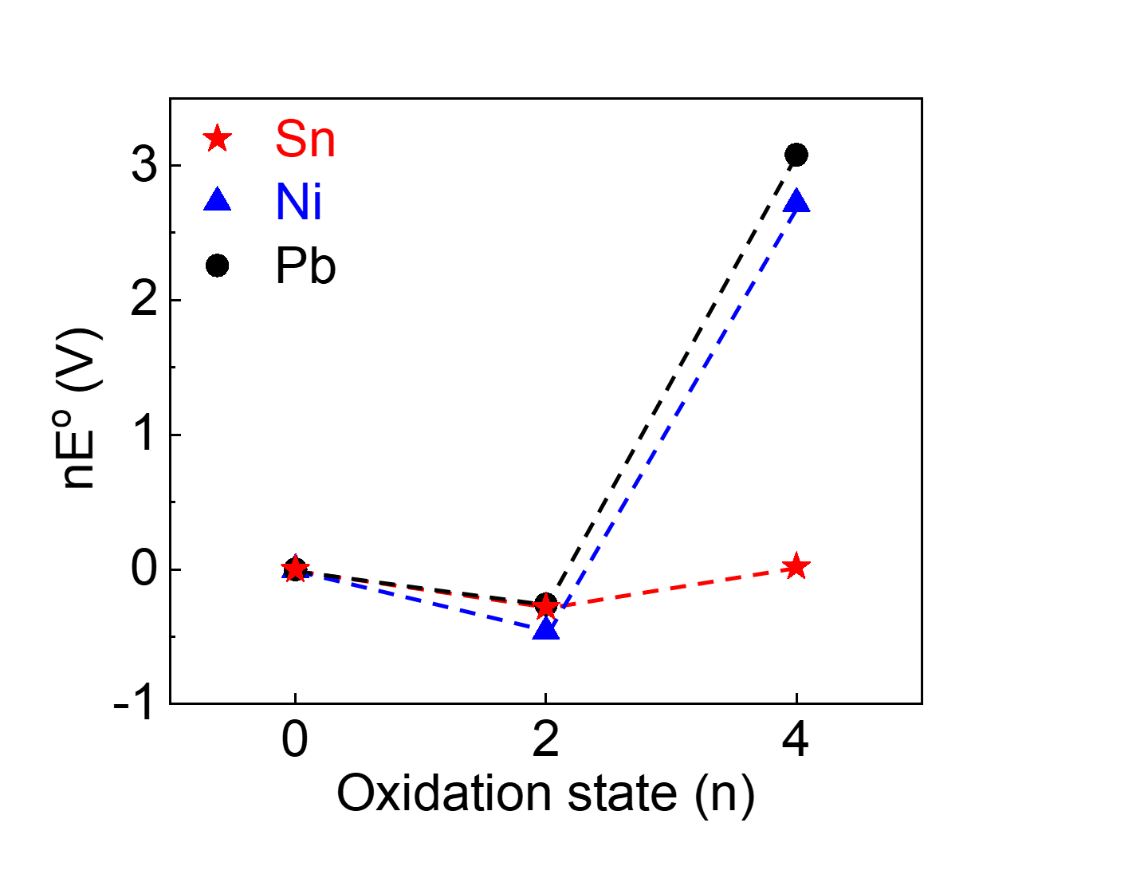


**Figure S8.** Frost diagrams of Sn, Ni, and Pb in different oxidation states derived from standard reduction potentials.


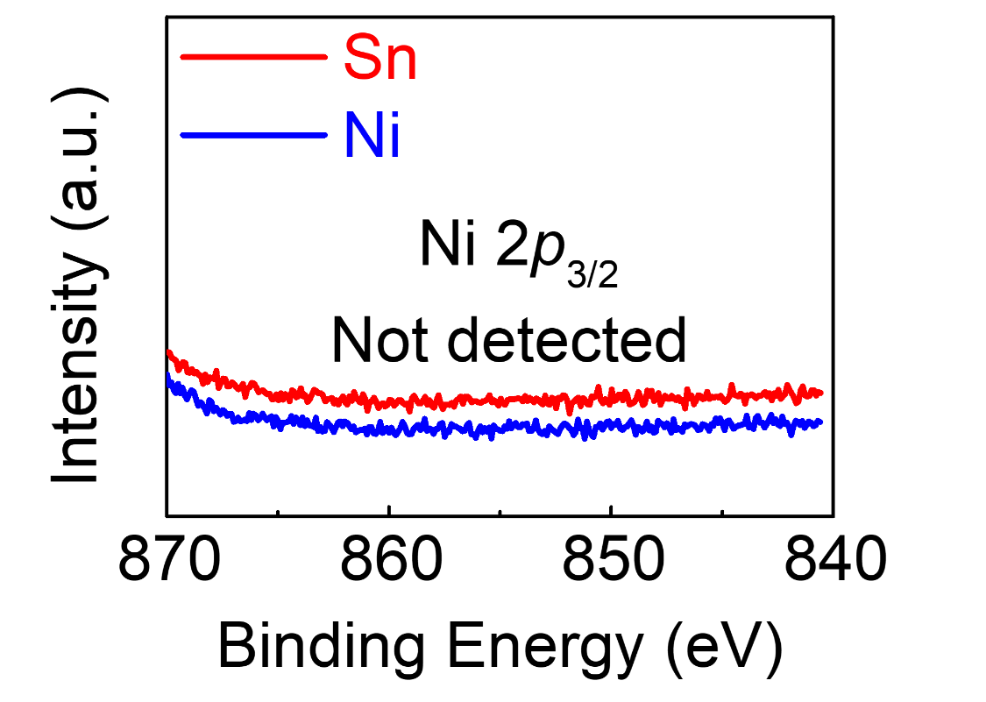


**Figure S9.** Ni 2*p*_3/2_ peak region from X-Ray photoelectron spectroscopy analysis of perovskite film prepared using Ni as a reducing agent.


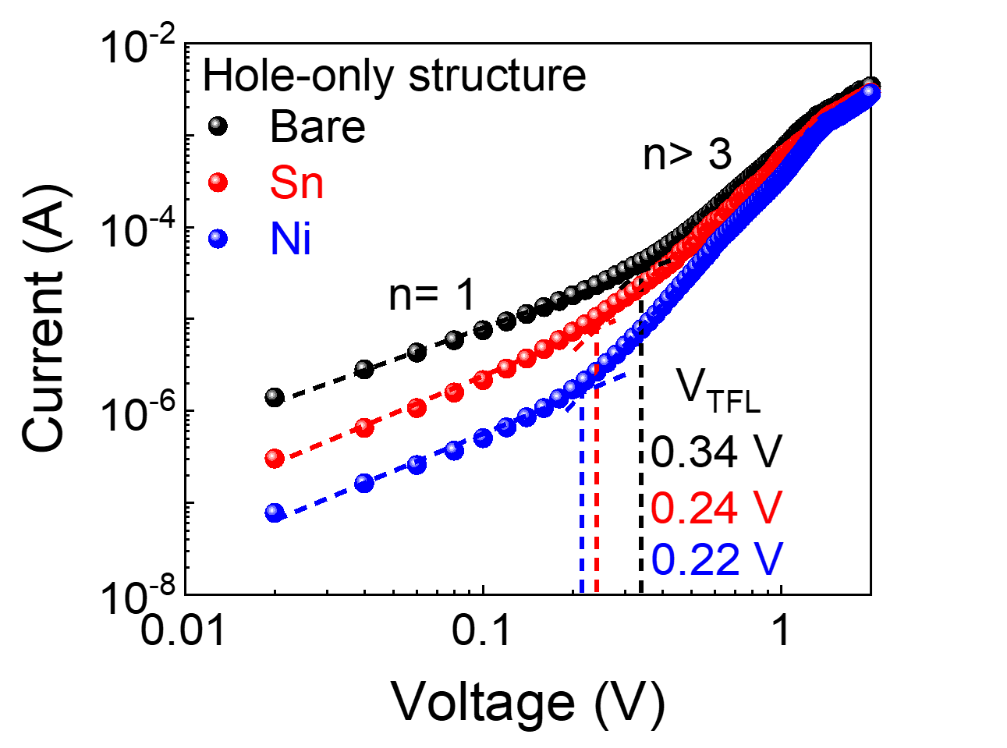


**Figure S10.** Space charge–limited current measurement of hole-only-structured device (FTO/PEDOT:PSS/perovskite/Spiro-OMeTAD/Au) based on Bare, Sn, and Ni samples.

**
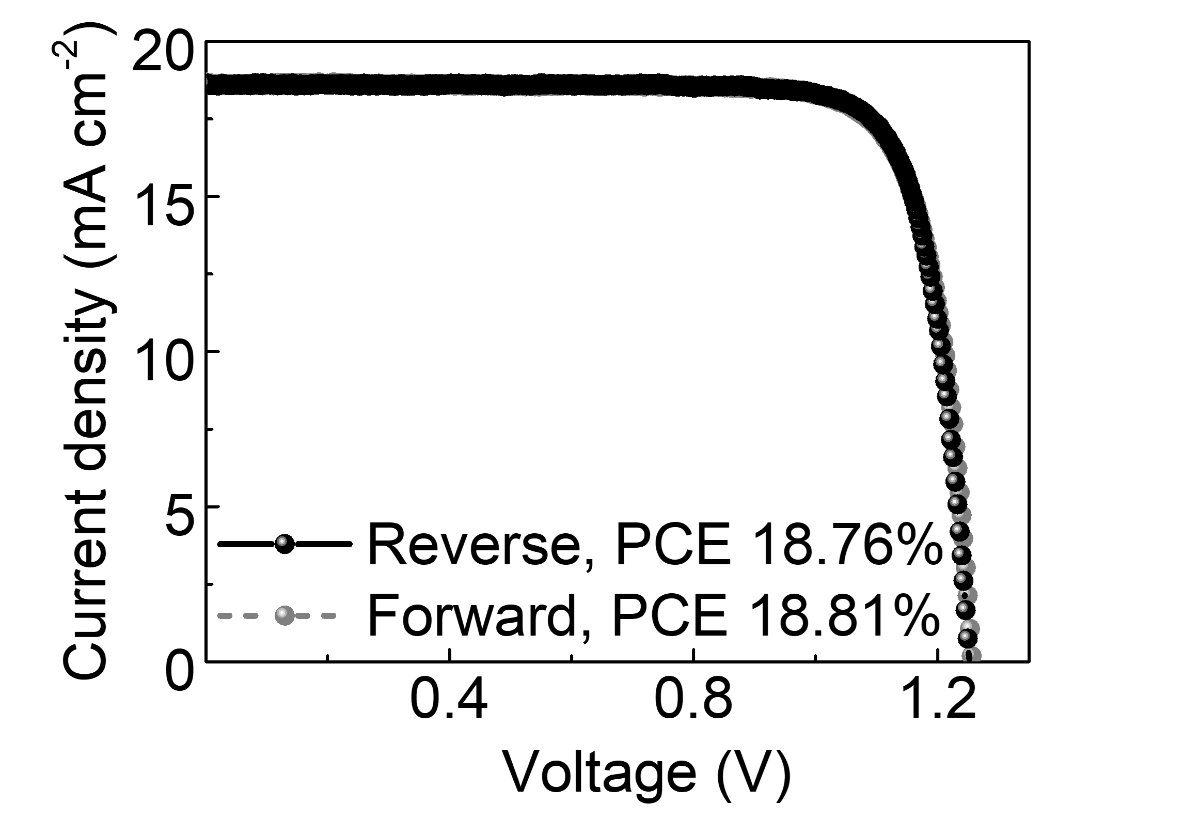
**

**Figure S11.** *J–V* curve of best-performing single-junction WBG device.

**
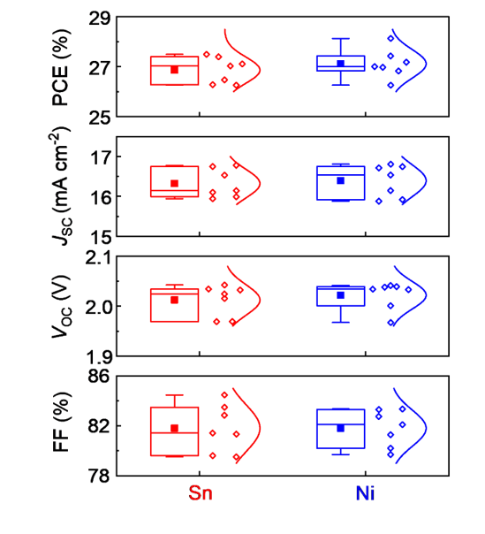
**

**Figure S12.** Statistical distributions of PV parameters of all-perovskite tandem solar cells using Sn and Ni as reductant.

**Table S1.** Half-reaction and standard reduction potentials of Sn, Ni, and Pb. ^[2]^

| Half reaction | E^o^ [V] |
| --- | --- |
| Pb^4+^ + 2e^-^ 🡪 Pb^2+^ | 1.67 |
| NiO_2_ (s) + 2H^+^ + 2e^-^ 🡪 Ni^2+^ + 2OH^-^ | 1.59 |
| Sn^4+^ + 2e^-^ 🡪 Sn^2+^ | 0.15 |
| Pb^2+^ + 2e^-^ 🡪 Pb^0^ | −0.13 |
| Sn^2+^ + 2e^-^ 🡪 Sn^0^ | −0.14 |
| Ni^2+^ + 2e^-^ 🡪 Ni^0^ | −0.23 |

**Table S2.** The decay lifetimes (τ_1_, τ_2_, and τ_3_) and effective (i.e. average) lifetime of the photo-generated charges in Bare, Sn, and Ni perovskite films, determined using TRPL decay curves with triexponential components. τ_average_ = (A_1_τ_1_ + A_2_τ_2_ + A_3_τ_3_ )/(A_1_ + A_2_ + A_3_).

|  | A_1_  (%) | τ_1_  (ns) | A_2_  (%) | τ_2_  (ns) | A_3_  (%) | τ_3_  (ns) | τ_average_  (ns) |
| --- | --- | --- | --- | --- | --- | --- | --- |
| Bare | 56.25 | 1.10 | 24.43 | 22.75 | 19.32 | 159.77 | 37.04 |
| Sn | 42.74 | 3.56 | 30.65 | 33.81 | 26.61 | 225.79 | 71.97 |
| Ni | 35.29 | 6.08 | 31.77 | 50.75 | 32.94 | 262.40 | 104.70 |

**Table S3.** Average and best device performance for single-junction WBG PSCs.

|  | *J*_SC_  [mA cm^-2^] | *V*_OC_  [V] | FF  [%] | PCE  [%] |
| --- | --- | --- | --- | --- |
| Average | 17.93 ± 0.06 | 1.244 ± 0.005 | 82.85 ± 2.51 | 18.48 ± 0.16 |
| Best  (Reverse scan) | 18.05 | 1.252 | 83.04 | 18.76 |
| Best  (Forward scan) | 18.07 | 1.255 | 82.95 | 18.81 |

**References**

[1] R. K. Gunasekaran, J. Jung, S. W. Yang, D. Im, W. C. Choi, Y. Yun, S. Lee, *ACS Energy Letters* **2024**, *9*, 102. https://doi.org/10.1021/acsenergylett.3c02402.

[2] J. D. P. Peter William Atkins, James Keeler, *Physical Chemistry*, 11th ed., Oxford University Press, **2018**.
